# Evaluation of the Validity and Reliability of the Chinese Healthy Eating Index

**DOI:** 10.3390/nu10020114

**Published:** 2018-01-24

**Authors:** Ya-Qun Yuan, Fan Li, Han Wu, Ying-Chuan Wang, Jing-Si Chen, Geng-Sheng He, Shu-Guang Li, Bo Chen

**Affiliations:** Key Laboratory of Public Health Safety of Ministry of Education, Collaborative Innovation Center of Social Risks Governance in Health, School of Public Health, Fudan University, Shanghai 200032, China; yqyuan16@fudan.edu.cn (Y.-Q.Y.); 16211020023@fudan.edu.cn (F.L.); 17211020098@fudan.edu.cn (H.W.); 17211020138@fudan.edu.cn (Y.-C.W.); 15211020022@fudan.edu.cn (J.-S.C.); gshe@shmu.edu.cn (G.-S.H.); leeshuguang@fudan.edu.cn (S.-G.L.)

**Keywords:** healthy eating index, validity, reliability, diet quality, China

## Abstract

The Chinese Healthy Eating Index (CHEI) is a measuring instrument of diet quality in accordance with the Dietary Guidelines for Chinese (DGC)-2016. The objective of the study was to evaluate the validity and reliability of the CHEI. Data from 12,473 adults from the China Health and Nutrition Survey (CHNS)-2011, including 3-day–24-h dietary recalls were used in this study. The CHEI was assessed by four exemplary menus developed by the DGC-2016, the general linear models, the independent *t*-test and the Mann–Whitney *U*-test, the Spearman’s correlation analysis, the principal components analysis (PCA), the Cronbach’s coefficient, and the Pearson correlation with nutrient intakes. A higher CHEI score was linked with lower exposure to known risk factors of Chinese diets. The CHEI scored nearly perfect for exemplary menus for adult men (99.8), adult women (99.7), and the healthy elderly (99.1), but not for young children (91.2). The CHEI was able to distinguish the difference in diet quality between smokers and non-smokers (*P* < 0.0001), people with higher and lower education levels (*P* < 0.0001), and people living in urban and rural areas (*P* < 0.0001). Low correlations with energy intake for the CHEI total and component scores (|r| < 0.34, *P* < 0.01) supported the index assessed diet quality independently of diet quantity. The PCA indicated that underlying multiple dimensions compose the CHEI, and Cronbach’s coefficient α was 0.22. Components of dairy, fruits and cooking oils had the greatest impact on the total score. People with a higher CHEI score had not only a higher absolute intake of nutrients (*P* < 0.001), but also a more nutrient-dense diet (*P* < 0.001). Our findings support the validity and reliability of the CHEI when using the 3-day–24-h recalls.

## 1. Introduction

Diet quality is the nutritional adequacy of daily diet, as well as the conformance of dietary patterns with national dietary guidelines [1,2]. Studies have proved that high-quality diets are associated with a reduction in the risk of various health outcomes, including cardiovascular diseases, cancer, type 2 diabetes mellitus, and all-cause mortality [3,4]. Diet quality indices or scores, the measuring instrument of diet quality, are employed to summarize dietary intake into a single numeric variable, which addresses the limitations in the assessment of diet-disease relationships based only on a single food or a single nutrient [5].

A variety of indices on diet quality have been developed worldwide. A well-known one is the American Healthy Eating Index (HEI). After its initial establishment in 1995, it was revised twice in 2005 and 2010, along with the release of the Dietary Guidelines for Americans [6,7,8]. All of the original HEI, HEI-2005, and HEI-2010 have been examined explicitly for their validity and reliability. Satisfactory validity and reliability of the index accelerated its promotion worldwide. Up till now, Canada, Brazil, Australia, Thailand and China have adapted the HEI for their own populations based on local dietary guidelines [9,10,11,12]. Some of the adaptions have been evaluated [12,13]. Validated diet indices are used widely in the assessment of dietary status, nutrition interventions, and epidemiology research [14,15,16,17,18].

The Chinese Healthy Eating Index (CHEI) is the first instrument in China to evaluate the overall diet quality in accordance with the updated Dietary Guidelines for Chinese (DGC-2016) [19]. The CHEI comprises 17 components, 12 of which evaluate the adequacy of a diet, including (1) total grains; (2) whole grains and mixed beans; (3) tubers; (4) total vegetables; (5) dark vegetables; (6) fruits; (7) dairy; (8) soybeans; (9) fish and seafood; (10) poultry; (11) eggs; (12) seeds and nuts. The other five components assess the limitation of a diet, referring to dietary components that should be consumed in moderation for the Chinese population, including (1) red meat; (2) cooking oils; (3) sodium; (4) added sugars; (5) alcohol.

Higher intakes of adequacy components will receive higher scores, whereas higher intakes of limitation components will receive lower scores. Therefore, for all components and the total score, higher scores reflect better diet quality. All of the 17 components are summarized to obtain a total score, which has a maximum value of 100.

Good validity and reliability are the premises of the application and promotion of a dietary index. Therefore, the purpose of the current study is to examine the validity and reliability of the CHEI, involving content validity, six kinds of construct validity including the association with nutrient intakes, and three kinds of reliability.

## 2. Materials and Methods

### 2.1. Data Source

Data from the China Health and Nutrition Survey (CHNS) were used in the current study. The CHNS is a prospective, ongoing, open cohort study conducted since 1989, drawing samples using a multistage, random cluster method. Four major regions (Northeast China: Heilongjiang, Liaoning; East Coast: Shandong, Jiangsu; Central China: Henan, Hubei, Hunan; and the West: Guangxi, Guizhou) have been involved in the CHNS since 2000, which mainly accommodated all levels of socioeconomic development across China. Details of the CHNS are described elsewhere [20].

The dietary assessment used the data from three consecutive days’ 24-h recalls at an individual level. In addition, condiments, including oil and sodium intakes, were collected from household food inventory during the same three-day period. At the individual level, trained interviewers recorded types, amounts and the eating location of all foods at each meal (at home and away from home) in every 24-h period, using food models and pictures. Household food consumption was estimated by examining the daily changes of inventory, using a weighing and measuring technique as described in detail elsewhere [21]. Data from 12,473 adults (≥18 years old) of the 2011 survey were used in the present study.

### 2.2. Evaluation Plan

To test the content validity of the CHEI, we checked the set of the components against the key recommendations of the DGC-2016 to confirm that they were captured in the CHEI.

Seven analyses were conducted to test construct validity (Table 1). Firstly, we estimated the intakes of eight food groups by quintiles of the CHEI scores to tell whether a higher CHEI score was associated with a lower exposure to the confirmed dietary risk factors among the Chinese population. Secondly, we examined the exemplary menus representing a high-quality diet in the DGC-2016. The DGC-2016 set dietary intake goals and corresponding exemplary menus for adult men, adult women, the healthy elderly, young children, adolescents, pregnant and lactating women, respectively, to provide a diet reference and to help people of different age and gender groups check their own dietary consumption [22,23]. In our study, because the CHEI was not developed for the pregnant and lactating women, and that the menus for adolescents were only based on school lunch (not daily intake), the menus for these three groups were excluded from the analysis. Therefore, four sets of daily menus (for adult men, adult women, the healthy elderly, and young children) were selected and scored using the CHEI to check how well the index assesses the diets of people from groups with different age and gender (Supplemental Table S1). Thirdly, we tested the differences of the component and total scores between smokers and non-smokers, people with higher and lower education levels, and people living in urban and rural areas to check if the index could distinguish between groups with known differences in diet quality. Fourthly, correlations between component scores and energy intake were estimated to check if the CHEI assessed the diet quality independent of the diet quantity. Fifthly, we used a principal component analysis (PCA) to estimate the underlying structure of the CHEI and to test if the index had 1 or >1 dimensions for the variation observed in the data. Finally, two analyses were conducted to evaluate the association between the CHEI score and the daily intakes of nutrients. On one hand, we estimated the distribution of macronutrient and selected micronutrient intakes across quintiles of the CHEI score. On the other hand, we further examined the Pearson coefficients between the index and each of the nutrients.

For reliability (Table 1), we estimated the internal consistency, which is one type of reliability, to address the question of whether all the CHEI component scores measure the same underlying and multidimensional construct. To explore the inside relations of the index, we examined the inter-component correlations. Additionally, we estimated the correlations between each of the component scores and the total score (minus that component score) to determine which component impacted the total score most.

### 2.3. Statistical Analyses

The CHEI scores reported in the present study were based on 3-day–24-h dietary recalls. Means of selected food groups across quintiles of the CHEI score were tested using *P* for trend calculated with general linear models [24]. The independent *t*-test and the Mann–Whitney *U*-test [25] were used to compare the total CHEI score and the individual component scores, respectively, between different characteristics of the participants (smoking status, education levels and living areas). The Mann–Whitney *U*-test was used because the CHEI component scores were non-normally distributed. Correlations of the CHEI total and component scores and energy intake were estimated by Spearman’s correlation coefficient. A PCA [26] was performed to examine the dimensions of the CHEI. The Cronbach’s coefficient α was calculated to test the internal consistency of the index [12,27]. Medians and interquartile ranges (IQRs) were calculated for all macro and micro-nutrients. The trends of nutrient intakes across quintiles of the CHEI score were tested with general linear models. The Box–Cox transformation [28] was applied as necessary to improve the normality of the residuals of general linear models. After the test of normality, we examined the Pearson correlations between the CHEI score and each of the trans-nutrient intakes. 

The statistical analyses were performed with the use of SAS software version 9.3 (SAS Institute Inc., Cary, NC, USA) and SPSS software version 22.0 (SPSS Inc., Chicago, IL, USA). Two-side *P*-values < 0.05 were considered statistically significant.

## 3. Results

### 3.1. Validity

The content validity of the CHEI was confirmed in our previous study [19]. The CHEI components were identified based on the key recommendations of the DGC-2016, which confirmed that these key recommendations were represented in the CHEI (Supplemental Table S2). 

Higher CHEI scores (across quintiles) were associated with higher intakes of whole grains, dairy products, seeds and nuts, fruits, vegetables, and fish and seafood. Higher CHEI scores were also associated with lower intakes of refined grains and red meat (Table 2).

The CHEI total scores for the four exemplary sets of menus ranged from 91.2 to 99.8 (Table 3). Nearly all component scores were at the maximum level for the first three menus (adult men, adult women and the healthy elderly). Only sodium in the menu for adult women and in that for the healthy elderly obtained 9.7 and 9.1, respectively. In addition, red meat on the menu of adult men obtained 4.8. However, the menu for young children received relative low points for total score (91.2) and for component scores of total grains (2.8), whole grains and mixed beans (3.3), fish and seafood (3.3), red meat (3.7) and sodium (8.1). 

The test of the scores between groups with known differences of diet quality revealed that the nonsmokers had significantly higher numbers of both total scores and scores of seven components when compared to the smokers (Table 4). Adults with education year ≥9 had a significant higher total score and higher scores for 12 components when compared to the adults with education year <9. Similarly, the urban residents’ mean total score was significantly higher than that of the rural residents. In addition, 13 component scores were significantly higher for urban residents compared with those of rural residents.

Table 5 showed the Spearman correlations between the score and the energy intake for each component. Thirteen of the 17 CHEI components were significantly correlated with energy intake. Energy intake was positively correlated with total grains, soybeans, seeds and nuts, cooking oils, sodium and added sugars, but negatively correlated with tubers, total vegetables, fruits, dairy, fish and seafood, eggs and alcohol. The strongest correlation lied in between energy intake and sodium scores, but the correlation coefficient was only 0.34. Additionally, the total score also had a low coefficient of positive correlation with energy intake (0.10). We therefore can conclude that all of the total scores and each component score were independent of energy intake.

**Figure 1:**
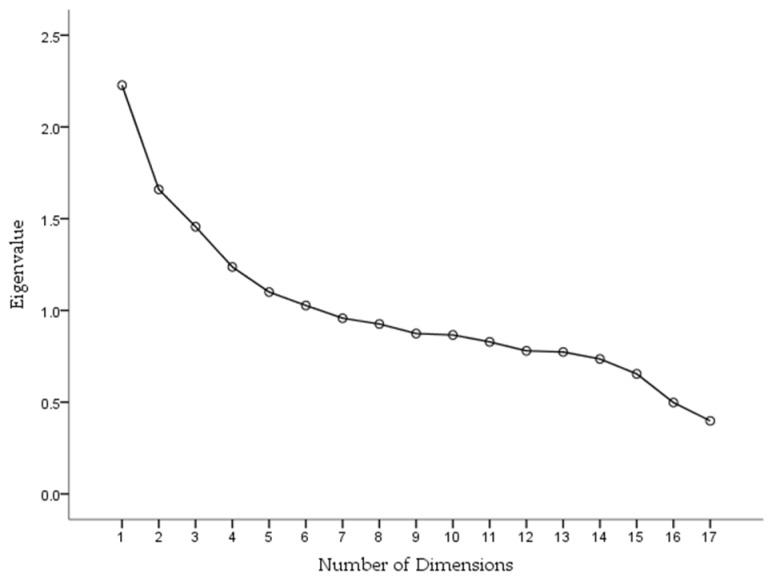
Scree plot from the principal component analysis of the CHEI showing the amount of variance accounted by each of the principal dimensions.

The CHEI scree plot revealed that multiple factors underline the index (Figure 1). The connecting curve between the dots became flat starting from the fifth dimension. The scree plot also showed the presence of six factors with eigenvalue >1, representing 51% of the total variance in the index. The factor-loading matrix for the six factors is shown in Supplemental Table S3. These results indicated that the CHEI had at least five dimensions.

**Figure 2:**
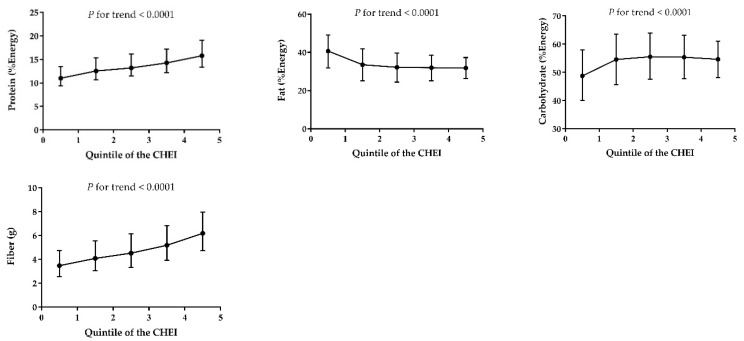
Median and interquartile range of the daily intake of macronutrients (per 1000 kcal) across each quintile of the CHEI scores for adults from the CHNS-2011.

**Figure 3:**
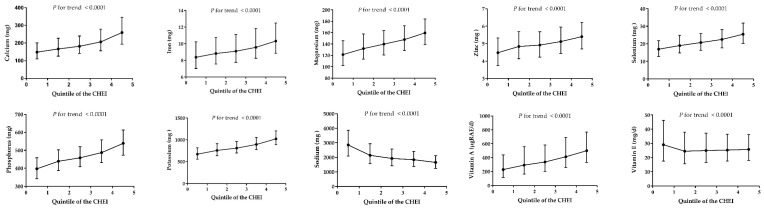

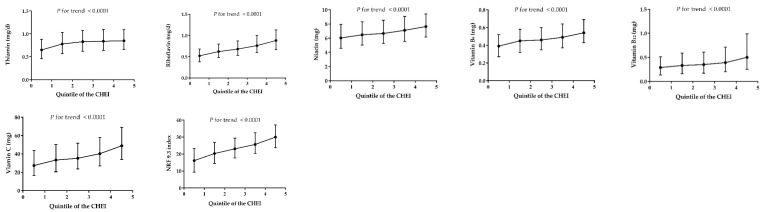
Median and interquartile range of daily intake of micronutrients (per 1000 kcal) across each quintile of the CHEI scores for adults from the CHNS-2011. The NRF9.3 index is the sum of the daily value for protein, dietary fibre, vitamin A, vitamin C, vitamin E, calcium, magnesium, iron, and potassium minus the daily value of saturated fat, added sugars, and sodium.

Figure 2 and Figure 3 shows the distribution of macronutrients and selected micronutrient intakes (after energy adjustment) across the CHEI quintile scores, respectively. The CHEI score was positively associated with protein, carbohydrate and fiber, but negatively associated with fat (*P* for trend <0.0001). For all the minerals except sodium, significant positive trends (*P* < 0.0001) were observed across quintiles of the CHEI score. Of the vitamins, only vitamin E showed an inverse association with the CHEI; the other vitamins were all positively associated with the increase of the CHEI. The results of nutrients distribution, as shown in Supplemental Figure S1, were consistent before and after the energy adjustment. Additionally, the increasing CHEI score was also linked with a higher score of the Nutrient-Rich Foods (NRF) 9.3 index, which was a tool to measure the nutritional quality of diets [29].

The relationship between the CHEI score and the level of nutrient intake was confirmed by the Pearson’s correlation (Table 6). After the energy adjustment, the CHEI demonstrated significant correlations with all macronutrients and micronutrients. The strongest correlations were protein (*r* = 0.420, *P* < 0.0001) among macronutrients, potassium (*r* = 0.492, *P* < 0.001) among minerals, and riboflavin (*r* = 0.455, *P* < 0.0001) among vitamins.

### 3.2. Reliability

The CHEI had a standard and nonstandard Cronbach’s coefficient α of 0.22 and 0.33, respectively. Generally, the inter-component correlations were quite low (Table 5). They ranged from −0.33 for dairy and added sugars to 0.46 for total vegetables and dark vegetables. Correlations between the score of certain components and the combined score of others ranged from −0.20 for added sugars and 0.27 for fruits. Four components had scores negatively related to the total score (total grains −0.10, red meat −0.13, added sugar −0.20, and alcohol −0.03), and the others had scores positively related to the total score.

## 4. Discussion

The evaluation results of this study supported the reliability and validity of the CHEI as an instrument for assessing the diet quality of the Chinese population based on the 3-day–24-h dietary recalls. 

Construct validity quantitatively estimates how well an index measures what it is expected to measure [30], which in the CHEI refers to the diet quality specified by the DGC-2016. According to the study on Global Burden of Diseases (GBD), the leading risk factor in China was a composite of 14 dietary factors [31]. Among them, seven factors, that are based on food groups, were employed in our study to conduct the first analysis of construct validity. Our results showed that all of these seven factors were significantly associated with the CHEI. Although the high intake of refined grains was not listed as one of the 14 risk factors in the GBD study [31], it has been identified as a strong risk factor of diabetes among the Chinese population [32] and was therefore incorporated into our analysis. Our results provided evidence that a higher CHEI score was linked with a lower exposure to known risk factors among Chinese diets.

Construct validity of a diet index should be supported by the analyses of exemplary menus, which demonstrated the ability of the index to capture the theoretical construct of a high-quality diet [27]. To evaluate the HEI-2010, the authors calculated the index with menus based on the USDA Food Patterns, the Dietary Approaches to Stop Hypertension (DASH) Eating Plan, the Harvard Medical School’s Healthy Eating Pyramid, and the AHA No-Fad Diet. It might be inappropriate to use these menus in our study as they were not recommended for the Chinese population. Instead, four sets of daily menus developed by the DGC-2016 were employed to test the suitability of the CHEI for people of different age and gender groups, since they represented the high-quality diets. The menus for adult men, adult women and the healthy elderly received perfect scores for nearly all components of the CHEI, suggesting a good fix of the index to the recommended menus of these people. However, the menu for young children did not receive full points in 5 of the 17 CHEI components, and the total score was only 91.2 points. This might be because the CHEI was developed based on the 1600–2400 kcal energy level, which did not capture the energy level of 1300 kcal for the menu of young children (Supplemental Table S1). Additionally, young children have different dietary requirements and behaviors compared to adults [33]. It might be necessary to modify the current CHEI to assess the diet quality of young children in China. 

Epidemiologic studies showed that diet quality is affected by socioeconomic status (SES) and lifestyle factors [34]. Our results showed that non-smokers, people with higher education levels or living in urban areas had a better diet quality than smokers, people with lower education levels and people living in rural areas, demonstrating the concurrent criterion-related validity of the CHEI. Unlike the evaluation of HEI-2005 and HEI-2010, we did not compare the CHEI scores between different age and gender groups. This was because the dietary data used in the present study were collected based on the household level, suggesting that strong correlations might exist among individuals from the same household.

The relatively low correlations between items of each component score, total scores, and energy intake indicated that the CHEI uncouples diet quality and diet quantity, which is similar to the situation in other established indices of dietary quality [12,27,30]. The scree plot from the PCA illustrated that no single linear combination of the 17 components explained a significant amount of variation in the data. This demonstrated the multidimensional nature of the CHEI for being capable of reflecting the complexity of diet patterns and for the reason that no one single component drove the total score. 

A high-quality diet should also be a nutrient-dense diet [35]. Because the CHEI was based on food groups, we used nutrient intakes as alternative measures of a healthy diet to confirm the construct validity. For macronutrients, more favorable CHEI scores were associated with higher intakes of protein, carbohydrate, dietary fiber and lower fat intake, both with and without the energy adjustment. For micronutrients, the increasing CHEI score was associated with higher intakes of calcium, iron, magnesium, zinc, selenium, phosphorus, potassium, vitamin A, thiamine, riboflavin, niacin, vitamin B-6, vitamin B-12, and vitamin C, either before or after the energy adjustment. These results verified our hypothesis that participants with a higher CHEI score had not only a higher absolute intake of these micronutrients, but also a relatively more nutrient-dense composition of the diet. The positive association between the CHEI and multiple nutrients was similar to other studies [24,36,37]. Understandably, sodium intake showed an inverse association with the CHEI score since sodium was regarded as a limitation component in the development of the index (a higher intake obtained a lower score). Vitamin E was also inversely associated with the CHEI score. This may be because the main source of vitamin E was plant oil, which was over-consumed among the Chinese population and also regarded as a limitation component of the CHEI. Given that the CHEI was developed based on food groups, and nutrients were not included in the calculation of the index, the positive relationship observed between a higher intake of nutrients and a higher score on the index support the validity of the CHEI. 

Additionally, the CHEI scores were positively related to the NRF 9.3 index, which was constructed to measure the nutritional quality of diets [29]. The CHEI and the NRF 9.3 are two independent indexes that reflect the food pattern and nutrient profiling of a diet, respectively. The positive correlation between the two independently developed scores confirmed the validity of the CHEI.

The standard and nonstandard Cronbach’s coefficient α of the CHEI were 0.22 and 0.33, which were lower than the commonly accepted standard of 0.70 for the internal consistency of an index. However, the internal consistency of a diet index is neither expected nor desired. Reasons for this could be that diet quality is known to have a multidimensional and complex construct and that people do not meet or fail to meet the dietary recommendations at the same time [30]. Dietary indexes developed in other countries had the Cronbach’s coefficient α varying from 0.28 to 0.68 [8,12,15,27,30]. Accordingly, the Cronbach’s coefficient of the CHEI is acceptable, indicating that more information and differences in diet quality can be determined by examining the total score as well as the component scores.

Correlations between components and the total score implied that the variance of each component contributes to the total score [27]. Components that showed higher correlations with the total score would reflect more variation. The correlations between component scores and the total score were generally quite low for the CHEI. Fruits, dairy and cooking oils were the ones that impacted the total score most. This suggested that fruit, dairy and cooking oil consumption exerted the greatest influence on the whole diet quality of the Chinese population; in other words, increasing the intakes of fruits and dairy, and decreasing the consumption of cooking oils could be the most important areas to target improvements in the diet quality of the Chinese population. Components such as tubers, alcohol, and soybeans had low correlations with the total score, indicating that they might not add much information to the total score, but rather provide independent information. 

Most components were positively associated with the total score. Only total grains, red meat, added sugar and alcohol had low and negative correlations with the total score. The relatively high correlation of dairy component with the total grains (−0.3), added sugars (−0.33) and red meat (−0.10) might be the main reason why they were negatively correlated with the total score. The alcohol component had very low correlations with any other components, which was probably because the 3-day average intake of alcohol was too low to represent a long-term consumption. 

The highest correlation was between total vegetables and dark vegetables, which was logical because dark vegetables are part of the total vegetables, and they are naturally correlated. Total grains had a relatively high correlation (0.33) with red meat, implying that people who consumed lower red meat were more likely to consume more total grains. This phenomenon was also confirmed by the positive correlation between whole grains and red meat. Another high correlation was between fruits and dairy, indicating that the protective diet factors of diets high in dairy and fruits often occurred consistently among the Chinese population.

To our knowledge, this is the first study to estimate the validity and reliability of a diet quality index in China. The strengths of this study include a large study population, comprehensive evaluation of the index involving content validity, various aspects of the construct validity and reliability. The correlation between two independent developed scores (the CHEI and the NRF9.3 index) is another strength of the study.

Our study was subject to some limitations. Firstly, the findings were based on the 3-day–24-h recalls, which cannot represent the average intake over a long period of time (usual intake) for an individual or a population. This might be the most significant limitation of the study. Because neither within-person nor between-person variability are adequately taken into account, the average intake over a small number of days cannot be an estimator of long-term usual intake [38]. The 3-day–24-h recall method is prone to underestimating the usual consumption, especially for episodically consumed foods [39] including alcohol, tubers, and seeds and nuts in our study. Therefore, further research in the future is needed to estimate the validity and reliability of the CHEI based on the usual intake of the Chinese population. Secondly, we did not compare the CHEI score with the gold standard such as blood nutrient concentration and nutritional biomarkers, to estimate nutritional status. Thirdly, the ability of the CHEI to predict diseases and death was not assessed. The index should be linked with longitude data containing health outcomes in a future study. Additionally, the CHNS-2011 had 1289 kinds of food, and only 567 of them contained saturated fat data in the database of China Food Composition [40,41]. This led to the underestimated calculation of saturated fat in our study. Finally, although the sample size was relatively large, data of the CHNS cannot represent the national status of dietary intake.

## 5. Conclusions

The CHEI has good validity and reliability for assessing the diet quality of the Chinese population based on the 3-day–24-h dietary recalls in the CHNS-2011.

## Figures and Tables

**Table 1:** Evaluation plans for the validity and reliability of the Chinese Healthy Eating Index (CHEI).

Evaluation Item	Analysis Question	Analysis Strategy
**Validity**		
Content validity	Does the CHEI catch the various key aspects of diet quality specified in the Dietary Guidelines for Chinese (DGC)-2016?	Compared the CHEI components against the key recommendations of the DGC-2016.
Construct validity	Does a higher CHEI score link with a lower exposure to confirmed dietary risk factors among the Chinese population?	Estimated the intakes of selected food groups by quintiles of the CHEI scores.
	Does the index give high scores to recommended menus for different gender and age groups?	Computed scores for exemplary menus developed for adult men, adult women, the healthy elderly, and young children.
	Does the index distinguish between groups with known differences in diet quality?	Compared component and total scores of smokers and non-smokers, people with higher and lower education levels and people living in urban and rural areas.
	Does the index assess diet quality independent of diet quantity?	Estimated the correlations between component scores and energy intake.
	How many dimensions does the CHEI have?	Used a principal components analysis to estimate the underlying structure of the index.
	What are the associations between the index and nutrient intakes?	Compared nutrient intakes across the quintiles of the CHEI score and examined the Pearson correlations between the index and each of the nutrients.
**Reliability**		
Internal consistency	How is the internal consistency of the index?	Estimated the Cronbach’s coefficient α.
	What are the correlations among the CHEI components?	Estimated Spearman correlations between each of the component scores.
	Which component has the biggest impact on the total score?	Estimated Spearman correlations between each component and the sum of all others.

**Table 2:** Means of selected foods by quintiles of the CHEI scores for adults in the 2011 China Health and Nutrition Survey (CHNS) according to the data of 3-day–24-h recalls, CHNS-2011.

	Quintile of the CHEI					
Selected Food Groups	Q1 (*n* = 2494)	Q2 (*n* = 2495)	Q3 (*n* = 2495)	Q4 (*n* = 2495)	Q5 (*n* = 2494)	*P* for Trend ^1^
CHEI score	38.02	46.89	52.45	58.21	68.01	
Whole grains (g/1000 kcal per day)	7.54	9.69	12.58	13.21	16.53	<0.0001
Refine grains (g/1000 kcal per day)	208.23	209.50	203.13	197.06	179.48	<0.0001
Dairy products (g/1000 kcal per day)	4.05	6.89	9.58	18.08	41.11	<0.0001
Seed and nuts (g/1000 kcal per day)	0.86	1.20	2.20	3.05	5.54	<0.0001
Fruits (g/1000 kcal per day)	7.81	15.08	27.30	44.75	79.07	<0.0001
Vegetables (g/1000 kcal per day)	145.48	154.22	156.78	159.96	167.43	<0.0001
Fish and seafood (g/1000 kcal per day)	7.70	9.85	11.58	14.64	21.98	<0.0001
Red meat (g/1000 kcal per day)	39.69	41.88	39.41	40.71	38.22	0.039

Q1–Q5, quintiles of the CHEI. ^1^ *P* for trend was calculated using general linear models. CHEI: Chinese Healthy Eating Index.

**Table 3:** The component scores and total scores of the CHEI for four menus recommended by the Dietary Guidelines for Chinese-2016.

	Exemplary Menus *			
CHEI Component (Maximum Score)	Adult Women ^1^	Adult Men ^2^	The Healthy Elderly ^3^	Young Children ^4^
Total grains (5)	5.0	5.0	5.0	2.8
Whole grains and mixed beans (5)	5.0	5.0	5.0	3.3
Tubers (5)	5.0	5.0	5.0	5.0
Total vegetables (5)	5.0	5.0	5.0	5.0
Dark vegetables (5)	5.0	5.0	5.0	5.0
Fruits (10)	10.0	10.0	10.0	10.0
Dairy (5)	5.0	5.0	5.0	5.0
Soybeans (5)	5.0	5.0	5.0	5.0
Fish and seafood (5)	5.0	5.0	5.0	3.3
Poultry (5)	5.0	5.0	5.0	5.0
Eggs (5)	5.0	5.0	5.0	5.0
Seeds and nuts (5)	5.0	5.0	5.0	5.0
Red meat (5)	5.0	4.8	5.0	3.7
Cooking oils (10)	10.0	10.0	10.0	10.0
Sodium (10)	9.7	10.0	9.1	8.1
Added sugars (5)	5.0	5.0	5.0	5.0
Alcohol (5)	5.0	5.0	5.0	5.0
Total score ^5^ (100)	99.7	99.8	99.1	91.2

* Details of the four menus are shown in the Supplemental Table S1. ^1^ Based on a 1-day sample menu for adult women having a light level of physical activity (PAL = 1.5, energy = 1800 kcal). ^2^ Based on a 1-day sample menu for adult men having a middle level of physical activity (PAL = 1.75, energy = 2400 kcal). ^3^ Based on a 1-day sample menu for the healthy elderly above 65 years old (energy = 1700 kcal). The definition of the healthy elderly is consist with the Standard on Chinese healthy elderly (2013) [23]. ^4^ Based on a 1-day sample menu for children of 3–5 years old (energy = 1300 kcal). ^5^ Total score is the sum of the 17 component scores.

**Table 4:** The comparison of component scores and total scores of the CHEI by different groups of sex, smoking status, education years, and living area in adults aged ≥18 years from the CHNS-2011 ^1^.

CHEI Component	Smokers (*n* = 2604)	Non-Smokers (*n* = 9869)	Education Year < 9 years (*n* = 8364)	Education Year ≥ 9 years (*n* = 4109)	Rural Area (*n* = 7347)	Urban Area (*n* = 5126)
Total grains	4.55 ± 0.02	4.57 ± 0.01	4.64 ± 0.01	4.42 ± 0.01 **	4.72 ± 0.01	4.35 ± 0.01 **
Whole grains and mixed beans	0.89 ± 0.03	1.00 ± 0.02 *	0.95 ± 0.02	1.03 ± 0.02 **	0..95 ± 0.02	1.01 ± 0.02 **
Tubers	2.23 ± 0.04	2.23 ± 0.02	2.22 ± 0.02	2.30 ± 0.03 *	2.28 ± 0.03	2.19 ± 0.03
Total vegetables	3.77 ± 0.03	3.89 ± 0.01 **	3.87 ± 0.01	3.85 ± 0.02	3.77 ± 0.02	4.01 ± 0.02 **
Dark vegetables	2.47 ± 0.04	2.59 ± 0.02 *	2.50 ± 0.02	2.68 ± 0.03 **	2.28 ± 0.02	2.96 ± 0.03 **
Fruits	2.33 ± 0.06	2.99 ± 0.04 **	2.36 ± 0.04	3.86 ± 0.06 **	2.31 ± 0.04	3.61 ± 0.05 **
Dairy	0.60 ± 0.03	0.72 ± 0.02 **	0.42 ± 0.01	1.25 ± 0.03 **	0.26 ± 0.01	1.30 ± 0.03 **
Soybeans	2.38 ± 0.04	2.49 ± 0.02	2.36 ± 0.02	2.69 ± 0.03 **	2.31 ± 0.03	2.68 ± 0.03 **
Fish and seafood	1.60 ± 0.04	1.57 ± 0.02	1.35 ± 0.02	2.04 ± 0.03 **	1.30 ± 0.02	1.97 ± 0.03 **
Poultry	1.53 ± 0.04	1.54 ± 0.01	1.32 ± 0.02	1.98 ± 0.04 **	1.26 ± 0.02	1.92 ± 0.03 **
Eggs	2.40 ± 0.04	2.41 ± 0.02	2.17 ± 0.02	2.87 ± 0.03 **	2.13 ± 0.02	2.77 ± 0.03 **
Seeds and nuts	0.92 ± 0.04	1.02 ± 0.02	0.86 ± 0.02	1.28 ± 0.03 **	0.82 ± 0.02	1.24 ± 0.03 **
Red meat	2.76 ± 0.03	2.97 ± 0.02 *	3.11 ± 0.02	2.54 ± 0.03 **	3.16 ± 0.02	2.58 ± 0.02 **
Cooking oils	7.67 ± 0.07	7.49 ± 0.04 *	7.29 ± 0.04	8.01 ± 0.05 **	7.47 ± 0.04	7.62 ± 0.05 **
Sodium	6.63 ± 0.06	6.35 ± 0.03 **	6.21 ± 0.02	6.82 ± 0.05 **	6.36 ± 0.04	6.47 ± 0.05 *
Added sugars	4.41 ± 0.03	4.35 ± 0.02	4.51 ± 0.02	4.07 ± 0.03 **	4.59 ± 0.01	4.03 ± 0.03 **
Alcohol	4.61 ± 0.03	4.85 ± 0.01 **	4.77 ± 0.01	4.82 ± 0.01	4.75 ± 0.01	4.86 ± 0.01 **
Total score ^2^	51.74 ± 0.21	52.97 ± 0.01 **	50.88 ± 0.11	56.48 ± 0.17 **	50.74 ± 0.11	55.55 ± 0.16 **

^1^ Values are means ± standard errors. The independent *t*-test was used for the total CHEI score. The Mann–Whitney *U*-test was used for the score of individual components. * *P* < 0.01. ** *P* < 0.0001. ^2^ The total score is the sum of the 17 component scores.

**Table 5:** Spearman correlations of the CHEI component scores, total scores, and energy intake based on the 3-day–24-h dietary recalls for adults from the CHNS-2011.

CHEI Component	Total Grains	Whole Grains and Mixed Beans	Tubers	Total Vegetables	Dark Vegetables	Fruits	Dairy	Soybeans	Fish and Seafood	Poultry	Eggs	Seeds and Nuts	Red Meat	Cooking Oils	Sodium	Added Sugars	Alcohol	Total Score ^1^	Energy
Total grains	1																		
Whole grains and mixed beans	0.06 *	1																	
Tubers	−0.08 *	0.12 *	1																
Total vegetables	−0.13 *	−0.09 *	−0.05 *	1															
Dark vegetables	−0.18 *	−0.10 *	−0.16 *	0.46 *	1														
Fruits	−0.17 *	0.13 *	0.08 *	0.06 *	0.05 *	1													
Dairy	−0.30 *	0.10 *	0.04 *	−0.01	0.06 *	0.29 *	1												
Soybeans	−0.12 *	−0.02	0.07 *	0.01	−0.03	0.05 *	0.07 *	1											
Fish and seafood	−0.22 *	−0.08 *	−0.05 *	0.08 *	0.13 *	0.14 *	0.18 *	0.06 *	1										
Poultry	−0.13 *	−0.04 *	−0.08 *	0.01	0.14 *	0.10 *	0.12 *	−0.02	0.18 *	1									
Eggs	−0.17 *	−0.07 *	0.12 *	0.04 *	0.05 *	0.20 *	0.26 *	0.07 *	0.12 *	0.00	1								
Seeds and nuts	−0.17 *	0.12 *	0.05 *	−0.03 *	0.02	0.20 *	0.17 *	0.03 *	0.08 *	0.06 *	0.09 *	1							
Red meat	0.33 *	0.17 *	0.14 *	−0.08 *	−0.20 *	−0.04 *	−0.10 *	−0.01	−0.12 *	−0.12 *	0.00	0.01	1						
Cooking oils	0.24 *	0.05 *	0.03 *	−0.02	0.00	0.14 *	0.10 *	0.04 *	0.03 *	0.12 *	0.05 *	0.07 *	−0.14 *	1					
Sodium	0.05 *	0.02	−0.04 *	−0.02	0.03 *	0.08 *	0.10 *	0.04 *	0.04 *	0.06 *	−0.01	0.07 *	−0.13 *	0.28 *	1				
Added sugars	0.21 *	−0.03 *	−0.02	−0.01	−0.05 *	−0.17 *	−0.33 *	−0.05 *	−0.15 *	−0.12 *	−0.15 *	−0.18 *	−0.02	−0.04	0.03 *	1			
Alcohol	0.09 *	0.00	−0.02	0.04 *	0.07 *	0.04 *	0.05 *	−0.03 *	−0.02	−0.03	0.02	−0.05 *	−0.01	−0.06 *	−0.07 *	−0.04 *	1		
Total score ^1^	−0.10 *	0.10 *	0.04	0.08 *	0.07 *	0.27 *	0.25 *	0.06 *	0.13 *	0.10 *	0.15 *	0.15 *	−0.13 *	0.22 *	0.14 *	−0.20 *	−0.03 *	1	
Energy	0.10 *	0.06	−0.04 *	−0.13 *	−0.14	−0.03 *	−0.04 *	0.03 *	−0.03 *	0.02	−0.10 *	0.12 *	−0.01	0.14 *	0.34 *	0.05 *	−0.18 *	0.10 *	1

^1^ Total score minus the score of a specified component. * *P* < 0.01.

**Table 6:** Pearson correlations between the CHEI and the intake of different nutrients based on 3-day–24-h dietary recalls, CHNS-2011.

Nutrients	Pearson Correlations Model 2			
	Coefficent ^1^	*P*	Coefficent ^2^	*P*
Macronutrients				
Protein	0.354	<0.0001	0.420	<0.0001
Fat	−0.105	<0.0001	−0.243	<0.0001
Carbohydrates	0.164	<0.0001	0.155	<0.0001
Dietary fiber	0.391	<0.0001	0.408	<0.0001
Micronutrients				
Calcium	0.423	<0.0001	0.407	<0.0001
Iron	0.271	<0.0001	0.269	<0.0001
Phosphorus	0.369	<0.0001	0.489	<0.0001
Magnesium	0.322	<0.0001	0.366	<0.0001
Potassium	0.431	<0.001	0.492	<0.001
Sodium	−0.305	<0.001	−0.360	<0.001
Selenium	0.349	<0.0001	0.368	<0.0001
Zinc	0.244	<0.0001	0.263	<0.0001
Vitamin A	0.284	<0.0001	0.260	<0.0001
Vitamin E	−0.054	<0.0001	−0.119	<0.0001
Thiamine	0.241	<0.0001	0.248	<0.0001
Riboflavin	0.437	<0.0001	0.455	<0.0001
Niacin	0.246	<0.0001	0.221	<0.0001
Vitamin B-6	0.281	<0.0001	0.285	<0.0001
Vitamin B-12	0.214	<0.0001	0.199	<0.0001
Vitamin C	0.339	<0.0001	0.313	<0.0001
NRF 9.3 ^3^			0.397	<0.0001

^1^ Before energy adjustment (daily nutrients were measured in g). ^2^ After energy adjustment (daily nutrients were measured in g per 1000 kcal). ^3^ The Nutrient-Rich Foods (NRF9.3) index is the sum of the daily value for protein, dietary fiber, vitamin A, vitamin C, vitamin E, calcium, magnesium, iron, and potassium minus the daily value of saturated fat, added sugars, and sodium.

## Supplementary Materials

The following are available online at http://www.mdpi.com/2072-6643/10/2/114/s1, Supplemental Table S1a: Dietary consumption goal and one-day exemplary menu for adult women having light level of physical activity (PAL = 1.5, energy = 1800 kcal); Supplemental Table S1b: One-day exemplary menu for adult men having middle level of physical activity (PAL = 1.75, energy = 2400 kcal); Supplemental Table S1c: One-day exemplary menu for the healthy elderly above 65 years old (energy = 1700 kcal); Supplemental Table S1d: One-day exemplary menu for children of 3-5 years old (energy = 1300 kcal). Supplemental Table S2: Components and weighting of Chinese Healthy Eating Index (CHEI) mapped to the key recommendations of the Dietary Guidelines for Chinese-2016 (DGC-2016). Grades of the evidence (ranked as A, B and C) were based on the Food and Health Evidence Based Review. (This table was adapted from Table 1 in our previous paper published in Nutrients [19]). Supplemental Table S3: Factor-loading matrix for the six factors with eigenvalue >1 underline the CHEI. Supplemental Figure S1: Median and interquartile range of daily macronutrients intakes across quintiles of the Chinese Healthy Eating Index scores for adults in the China Health and Nutrition Survey-2011 (without energy adjustment).
