# Supplementary material for: Evaluation of the Validity and Reliability of the Chinese Healthy Eating Index
Source: Nutrients. 2018 Jan 24;10(2):114. doi: 10.3390/nu10020114 (PMC5852690; doi:10.3390/nu10020114)
Supplement: Supplementary file 1 [file nutrients-10-00114-s001.docx]

| **Supplemental Table 1a.** Dietary consumption goal and one-day exemplary menu for adult women having light level of physical activity (PAL=1.5, energy=1800 kcal) | | | |
| --- | --- | --- | --- |
| Dietary consumption goal | | | |
| Food groups | Goal intakes (gram) | Key suggestions | |
| Grains  Tubers | 225  50 | One third of the grains should be whole grains and mixed beans. | |
| Vegetables  Fruits | 400  200 | Choose a variety of vegetables and fruits, and half of the vegetables should be dark vegetables. | |
| Meat and poultry  Fish and seafood  Eggs | 50  50  40 | Prefer to fish and poultry, lean meat is better than fat meat.  Eat egg with yolk. | |
| Soy beans and products  Seeds and nuts  Dairy | 15  10  300 | Consume dairy every day.  Eat soy beans and product usually.  Eat seeds and nuts moderately. | |
| Cooking oils  Salt | 25  5 | Consume less salt and cooking oils and develop a light eating habit. | |
| One-day exemplary menu | | | |
| Dining | Food name | | Amount (gram) |
| [Breakfast](file:///C:\Users\Yaqun%20Yuan\AppData\Local\youdao\dict\Application\7.5.1.0\resultui\dict\?keyword=breakfast) | Oat | | 25 |
|  | Egg | | 40 |
|  | Milk | | 300 |
|  | Celery | | 50 |
|  | Peanut | | 10 |
| Lunch | Rice | | 100 |
|  | Foxtail millet | | 25 |
|  | [Chicken,](file:///C:\Users\Yaqun%20Yuan\AppData\Local\youdao\dict\Application\7.5.1.0\resultui\dict\?keyword=chicken)[wing](file:///C:\Users\Yaqun%20Yuan\AppData\Local\youdao\dict\Application\7.5.1.0\resultui\dict\?keyword=wing) | | 50 |
|  | [Spinage](file:///C:\Users\Yaqun%20Yuan\AppData\Local\youdao\dict\Application\7.5.1.0\resultui\dict\?keyword=spinage) | | 200 |
|  | Potato | | 100 |
|  | Egg | | 10 |
|  | Laver, dried | | 2 |
| Supper | Rice | | 75 |
|  | Perch | | 50 |
|  | Soybean curd, northern style | | 100 |
|  | Shiitake mushroom | | 10 |
|  | Rape | | 150 |
|  | Apple | | 200 |
| Condiments and other foods | Cooking oil | | 25 |
|  | Salt | | 5 |
|  | Alcohol | | < 15 |
|  | Added sugar | | < 25 |

| **Supplemental Table 1b.** One-day exemplary menu for adult men having middle level of physical activity (PAL=1.75, energy=2400 kcal) | | |
| --- | --- | --- |
| Dietary consumption goal | | |
| Food groups | Goal intakes (gram) | Key suggestions |
| Grains  Tubers | 300  100 | One third of the grains should be whole grains and mixed beans. |
| Vegetables  Fruits | 500  350 | Choose a variety of vegetables and fruits, and half of the vegetables should be dark vegetables. |
| Meat and poultry  Fish and seafood  Eggs | 75  75  50 | Prefer to fish and poultry, lean meat is better than fat meat.  Eat egg with yolk. |
| Soy beans and products  Seeds and nuts  Dairy | 25  10  300 | Consume dairy every day.  Eat soy beans and products usually.  Eat seeds and nuts moderately. |
| Cooking oils  Salt | 30  5 | Consume less salt and cooking oils and develop a light eating habit. |
| One-day exemplary menu | | |
| Dining | Food name | Amount (gram) |
| [Breakfast](file:///C:\Users\Yaqun%20Yuan\AppData\Local\youdao\dict\Application\7.5.1.0\resultui\dict\?keyword=breakfast) | Wheat flour | 25 |
|  | Bok-choy | 50 |
|  | Shiitake mushroom | 5 |
|  | Soybean curb slab | 20 |
|  | Egg | 40 |
|  | Milk | 300 |
|  | Apple | 150 |
| Lunch | Rice | 125 |
|  | Foxtail millet | 25 |
|  | [Chicken](file:///C:\Users\Yaqun%20Yuan\AppData\Local\youdao\dict\Application\7.5.1.0\resultui\dict\?keyword=chicken) | 50 |
|  | Chestnut | 15 |
|  | Garlic stalk | 100 |
|  | Pork | 25 |
|  | [Spinage](file:///C:\Users\Yaqun%20Yuan\AppData\Local\youdao\dict\Application\7.5.1.0\resultui\dict\?keyword=spinage) | 100 |
|  | Egg | 10 |
| Supper | Wheat flour | 75 |
|  | Corn flour | 50 |
|  | Clam | 75 |
|  | Soybean curd, soft, southern style | 75 |
|  | Green pepper | 50 |
|  | Potato | 100 |
|  | Carrot | 100 |
|  | Mung bean sprout | 100 |
|  | Banana | 200 |
| Condiments and other foods | Cooking oil | 30 |
|  | Salt | 5 |
|  | Alcohol | < 15 |
|  | Added sugar | < 25 |

| **Supplemental Table 1c.** One-day exemplary menu for the healthy elderly above 65 years old (energy=1700 kcal) | | |
| --- | --- | --- |
| Dietary consumption goal | | |
| Food groups | Goal intakes (gram) | Key suggestions |
| Grains  Tubers | 220  100 | Consume a cereal-based diet, one third of the grains should be whole grains and mixed beans.  Choose appropriate cooking methods. |
| Vegetables  Fruits | 350  200 | Eat sufficient and a variety of vegetables and fruits every day.  Half of the vegetables should be dark vegetables. |
| Meat and poultry  Fish and seafood  Eggs | 45  45  40 | Eat fish, poultry, lean meat and eggs usually. Ensure high quality protein consumption. |
| Soy beans and products  Seeds and nuts  Dairy | 15  10  300 | Moderately consume dairy and soybean products every day. |
| Cooking oils  Salt | 25  6 | Consume less salt and cooking oils and develop a light eating habit. |
| One-day exemplary menu | | |
| Dining | Food name | Amount in grams |
| [Breakfast](file:///C:\Users\Yaqun%20Yuan\AppData\Local\youdao\dict\Application\7.5.1.0\resultui\dict\?keyword=breakfast) | Wheat flour | 50 |
|  | Shiitake mushroom | 5 |
|  | Bok-choy | 50 |
|  | Egg | 30 |
|  | Soybean milk | 250 |
|  | Cheese | 20 |
| Lunch | Rice | 75 |
|  | Foxtail millet | 10 |
|  | Red bean | 25 |
|  | Green pepper | 100 |
|  | Potato | 100 |
|  | Cashew nut | 10 |
|  | Chicken, leg | 50 |
|  | Laver, dried | 2 |
|  | Egg | 10 |
| Supper | Rice | 50 |
|  | Long grained rice, purple | 25 |
|  | Yellow croaker, small | 50 |
|  | Soybean curd, northern style | 50 |
|  | [Spinage](file:///C:\Users\Yaqun%20Yuan\AppData\Local\youdao\dict\Application\7.5.1.0\resultui\dict\?keyword=spinage) | 200 |
|  | Pear | 100 |
| Extra meal | Pomelo | 200 |
|  | Milk | 300 |
| Condiments | Soybean oil | 25 |
|  | Salt | < 6 (5.5 g was used) |

| **Supplemental Table 1d.** One-day exemplary menu for children of 3-5 years old (energy= 1300 kcal). | | |
| --- | --- | --- |
| Dietary consumption goal | | |
| Food groups | Goal intakes (gram) | Key suggestions |
| Grains  Tubers | 100  25 | One third of the grains should be whole grains and mixed beans; Choose appropriate cooking methods. |
| Vegetables  Fruits | 250  150 | Choose a variety of vegetables and fruits, and half of the vegetables should be dark vegetables.  Eat fruits every day. |
| Meat and poultry  Fish and seafood  Eggs | 25  20  25 | Prefer to fish and poultry.  Consume lean meat instead of fat meat.  Eat egg with yolk. |
| Soy beans and products  Seeds and nuts  Dairy | 15  5  500 | Consume dairy every day, including milk, yogurt and cheese.  Eat soy beans and products usually.  Eat seeds and nuts moderately. |
| Cooking oils  Salt | 20  5 | Consume less salt and cooking oils and develop a light eating habit. |
| One-day exemplary menu | | |
| Dining | Food name | Amount in grams |
| [Breakfast](file:///C:\Users\Yaqun%20Yuan\AppData\Local\youdao\dict\Application\7.5.1.0\resultui\dict\?keyword=breakfast) | Oat | 10 |
|  | Rice | 10 |
|  | Walnut | 5 |
|  | Egg | 30 |
|  | Cheese | 10 |
| Lunch | Rice | 25 |
|  | Foxtail millet | 15 |
|  | [Chicken](file:///C:\Users\Yaqun%20Yuan\AppData\Local\youdao\dict\Application\7.5.1.0\resultui\dict\?keyword=chicken) | 25 |
|  | Shiitake mushroom | 10 |
|  | Broccoli | 100 |
|  | Potato | 50 |
| Supper | Rice | 45 |
|  | Pumpkin | 100 |
|  | Perch | 25 |
|  | Rape | 100 |
|  | Soybean curd | 100 |
|  | Pork, [minced](file:///C:\Users\Yaqun%20Yuan\AppData\Local\youdao\dict\Application\7.5.1.0\resultui\dict\?keyword=minced) | 30 |
| Extra meal | Banana | 150 |
|  | Milk | 250 |
|  | Yoghourt | 250 |
| Condiments | Cooking oil | 20 |
|  | Salt | 5 |

**Supplemental Table 2.** Components and weighting of Chinese Healthy Eating Index (CHEI) mapped to the key recommendations of the Dietary Guidelines for Chinese-2016 (DGC-2016). Grades of the evidence (ranked as A, B and C) were based on the Food and Health Evidence Based Review. (This table was adapted from Table 1 in our previous paper published in Nutrients [1])

| **Components** | **Weighting** | **Key Recommendations** | **Comments** | **Key Evidence** |
| --- | --- | --- | --- | --- |
| Total Grains  Whole Grains and Mixed Beans  Tubers | 5  5  5 | Eat a variety of foods, cereal-based.  Consume cereal at every meal.  Increase intake of whole grains and mixed beans.  Cook tubers in various forms to increase consumption. | Whole grains prevalent in China are coarse rice, whole wheat, corn, millet, buckwheat and oats.  Mixed beans are rich in carbohydrates, including mung bean, red bean and kidney bean, etc.  Tubers, such as potatoes, sweet potatoes and cassava, are also recommended as staple food for their high content of carbohydrates. | Consumption of whole grain reduces the risk of colorectal cancer, type 2 diabetes, cardiovascular disease and weight gain (B).  Increased tuber intake reduces the risk of constipation (C). |
| Total Vegetables  Dark Vegetables  Fruits  Dairy  Soybeans  Seeds and Nuts | 5  5  10  5  5  5 | Eat plenty of vegetables, fruits, dairy products, and soybeans.  Eat vegetables every meal, and half should be dark vegetables.  Every day consume fresh fruits rather than processed forms.  Consume a variety of dairy.  Consume soybeans regularly.  Seeds and nuts are beneficial, but should not be consumed excessively. | Dark vegetables include deep green, orange, red and fuchsia vegetables, such as spinach, tomatoes, carrots, and purple cabbage.  Cooked, canned, frozen, and dried fruits cannot replace fresh fruits.  Yogurt or low-lactose dairy products should be the first choice for individuals with lactose intolerance.  Although seeds and nuts are beneficial for health, excessive intake can lead to an excess of energy. | Total vegetable consumption reduces all causes of mortality and the risk of cardiovascular diseases (CVDs) and cancers of the digestive tract; intake of dark green vegetables lowers risks of type 2 diabetes and lung cancer (B).  Increased fruit intake lowers the risk of CVDs, cancers of the digestive tract and adult weight gain (B).  Low-fat milk consumption decreases the risk of breast cancer, and higher dairy intake is linked to higher bone mineral density (B).  Soybean intake lowers the risks of breast cancer, osteoporosis, type 2 diabetes, hyperlipidemia and hypertension (B).  Moderate intake of seeds and nuts decreases all causes of mortality and the risk of CVDs, hypertension, and colorectal cancer in women (B). |
| Fish and Seafood  Red Meat  Poultry  Eggs | 5  5  5  5 | Eat moderate amounts of fish, poultry, eggs, and lean meats.  Choose fish, seafood and poultry.  Decrease intake of fat meat and smoked meat products. | Adverse effects of excessively consuming red meat have been demonstrated.  Eat egg with yolk. | Consumption of fish lowers risk of CVDs, stroke (B), cognitive decline and macular degeneration (C).  Excessive intake of meat increases all-cause mortality in men and risk of type 2 diabetes, colorectal cancer (B), and obesity (C). Higher meat intake lowers risk of iron deficiency anemia (C). |
| Cooking Oils  Sodium  Added Sugars  Alcohol | 10  10  5  5 | Limit salt, cooking oil, added sugar, and alcohol.  Limit cooking oils intake to 25–30g/day.  Consume salt less than 6 g/day, and consume sodium less than 1500 mg/day.  Limit intake of added sugars to less than 50 g/day.  Children, adolescents, pregnant and lactating women should not consume alcohol; men (women) should limit alcohol intake to less than 25 g (15 g). | Cooking oils include plant oil and animal fat.  The sodium component of the CHEI also includes sodium in sodium-rich foods (sodium content more than 500 mg/100 g). | Excessive consumption of any kind of fat increases energy intake and the risk of obesity (A).  High consumption of sodium increases risk of hypertension (A), CVDs (C), stroke (B), gastric cancer (B).  Overconsumption of added sugar increases risk of dental caries (B), weight gain (C), and hyperlipidemia (C).  Excessive intake of alcohol rises risk of liver injury (A), gout (A), colorectal cancer (B), breast cancer (B), CVDs (B), and fetal alcohol syndrome (A). |

| Supplemental Table 3. Factor-loading matrix for the six factors with eigenvalue >1 underline the CHEI. | | | | | | |
| --- | --- | --- | --- | --- | --- | --- |
| **CHEI Component** | **Factor 1** | **Factor 2** | **Factor 3** | **Factor 4** | **Factor 5** | **Factor 6** |
| Total Grains | -0.430 |  | 0.480 | 0.366 | -0.110 | 0.336 |
| Whole grains and mixed beans | 0.143 |  | 0.620 |  |  |  |
| Tubers |  |  | 0.201 |  | 0.605 |  |
| Total Vegetables |  | 0.872 |  |  |  |  |
| Dark Vegetables |  | 0.805 | -0.160 |  | -0.180 |  |
| Fruits | 0.564 | 0.127 | 0.138 | 0.198 |  |  |
| Dairy | 0.680 |  | -0.108 |  |  |  |
| Soybeans |  |  | -0.241 | 0.101 | 0.585 |  |
| Fish and Seafood | 0.342 | 0.145 | -0.340 |  |  | -0.121 |
| Poultry | 0.251 |  | -0.199 | 0.140 | -0.468 | -0.228 |
| Eggs | 0.486 | 0.104 |  |  | 0.374 | 0.117 |
| Seeds and Nuts | 0.372 |  | 0.230 |  | -0.107 |  |
| Red Meat |  |  | 0.686 | -0.221 | 0.123 |  |
| Cooking Oils |  |  |  | 0.822 |  | -0.128 |
| Sodium |  |  | -0.129 | 0.692 |  |  |
| Added Sugars | -0.582 |  |  | 0.142 | 0.155 |  |
| Alcohol | 0.220 |  |  |  | -0.111 | 0.793 |

| 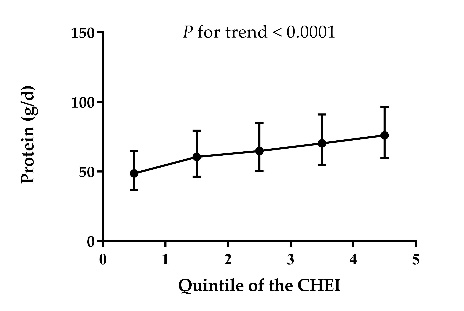 | 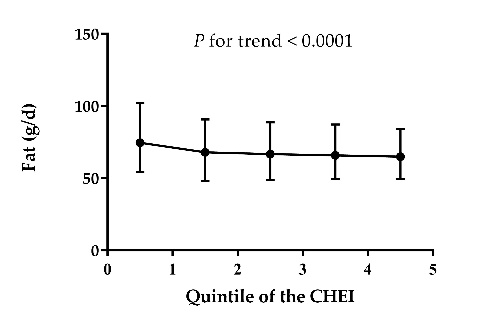 | 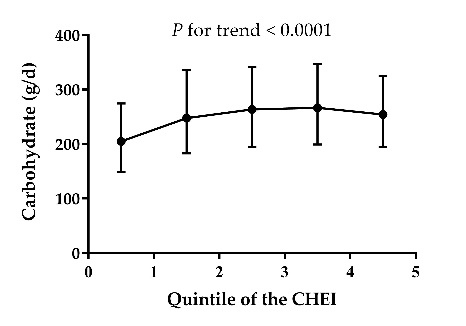 |
| --- | --- | --- |
| 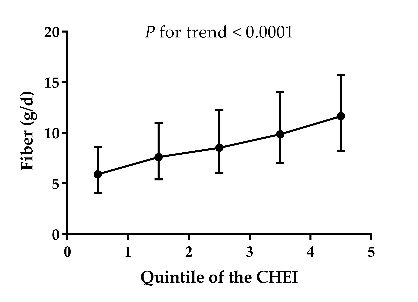 |  |  |
| **Supplemental Figure 1a.** Median and interquartile range of daily macronutrients intakes across quintiles of the Chinese Healthy Eating Index scores for adults in the China Health and Nutrition Survey-2011 (without energy adjustment). | | |

| 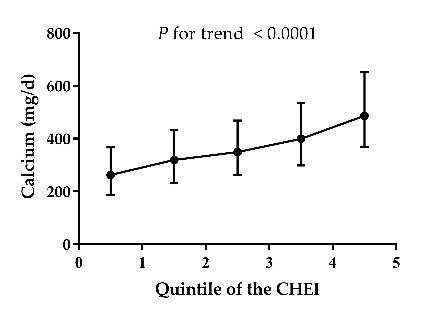 | 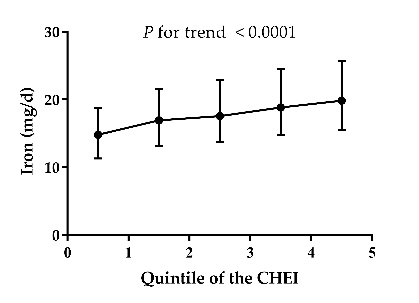 | 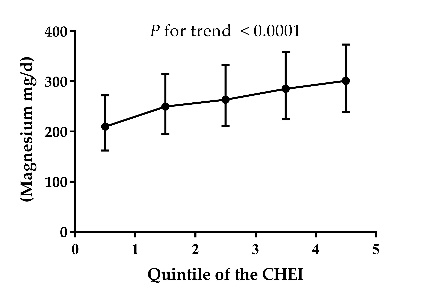 | 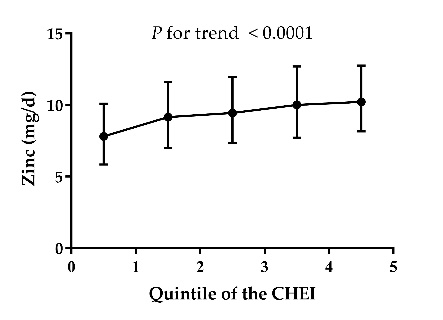 | 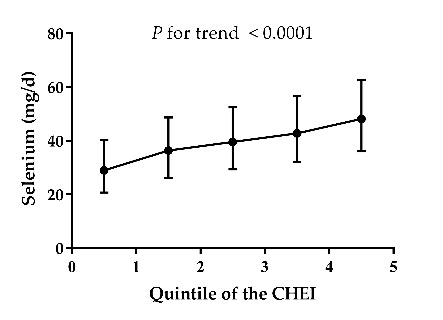 |
| --- | --- | --- | --- | --- |
| 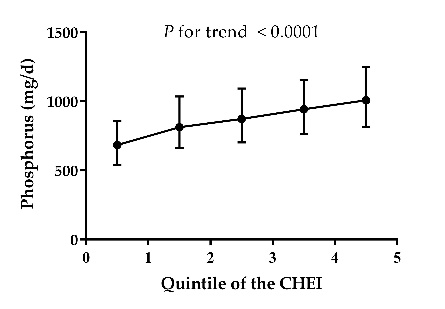 | 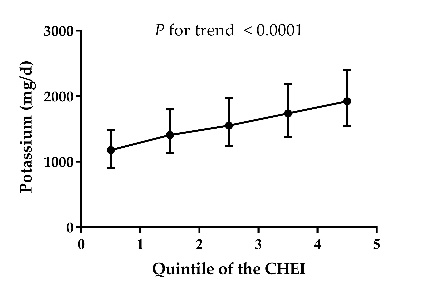 | 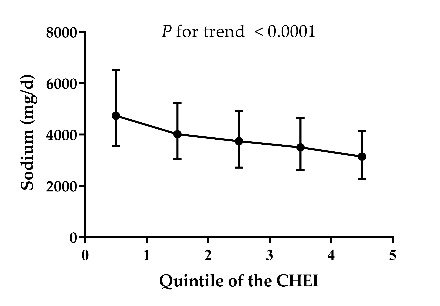 | 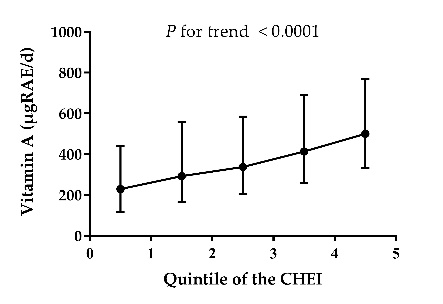 | 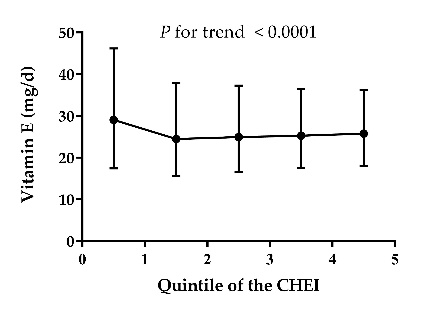 |
| 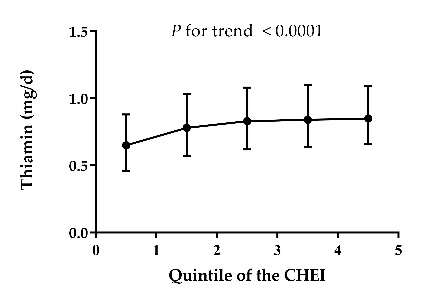 | 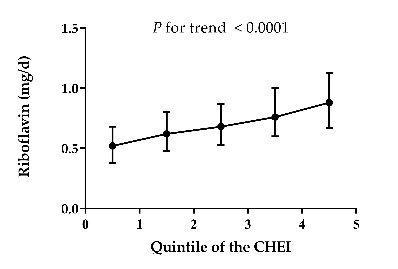 | 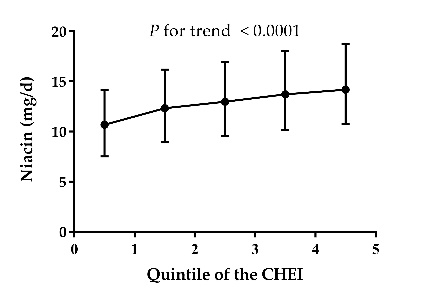 | 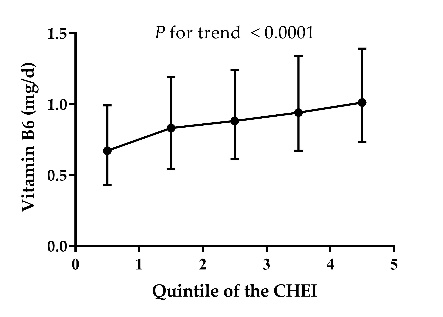 | 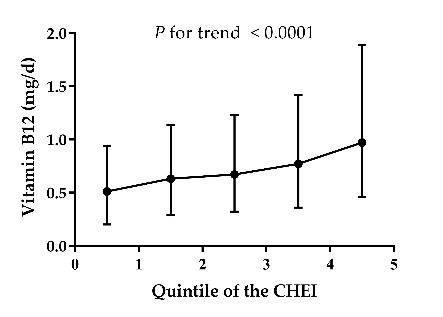 |
| 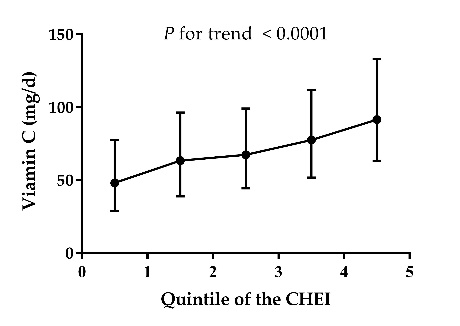 |  |  |  |  |
| **Supplemental Figure 1b.** Median and interquartile range of daily micronutrients intakes across quintiles of the Chinese Healthy Eating Index scores for adults in the China Health and Nutrition Survey-2011 (without energy adjustment). | | | | |

1. Yuan, Y.-Q.; Li, F.; Dong, R.-H.; Chen, J.-S.; He, G.-S.; Li, S.-G.; Chen, B. The development of a chinese healthy eating index and its application in the general population. *Nutrients* **2017**, *9*, 977.
